# The protection of sulforaphane on subarachnoid hemorrhage-induced intestinal mucosa injury in rats

**DOI:** 10.3389/fmolb.2025.1635795

**Published:** 2025-08-22

**Authors:** Zixiang Liu, Pengpeng Li, Yuanhai Zhang, Shidi Zhao, Wei Gao

**Affiliations:** ^1^ Department of Neurosurgery, Jiangnan University Medical Center, Wuxi, Jiangsu, China; ^2^ Department of Neurology, Wuxi Ninth People's Hospital Affiliated to Soochow University, Wuxi, Jiangsu, China

**Keywords:** SFN, SAH, Keap1/Nrf-2/HO-1 pathway, autophagy, intestinal mucosa injury

## Abstract

**Introduction:**

Sulforaphane (SFN) is recognized for its anti-inflammatory properties; however, the underlying molecular mechanisms remain unclear. In this study, we explored the effect of SFN on subarachnoid hemorrhage (SAH) and the potential mechanisms.

**Methods:**

Sprague–Dawley (SD) rats were divided into three groups (n = 12): Sham + vehicle group (Sham + V), SAH + vehicle group (SAH + V), and SAH + SFN group (SAH + S). SFN (50 mg/kg) dissolved in 250–280 μL corn oil was intraperitoneally injected, and the same volume of corn oil was served as the control. The appetite score, gut wet/dry weight ratio, and histological changes in ileum tissues were examined to determine intestinal mucosal injury. Quantitative real-time PCR (qRT-PCR) and Western blot were performed to examine the expression of genes. LC3 immunofluorescence and Hoechst 33258 staining were used to assess cell autophagy and apoptosis.

**Results:**

Compared to the SAH + V group, the SAH + S group demonstrated a significantly increased appetite score (1.55 ± 0.23 vs. 1.90 ± 0.35); decreased gut wet/dry weight ratio (4.02 ± 0.21 vs. 3.18 ± 0.21) and inflammatory score (2.89 ± 0.33 vs. 1.89 ± 0.60); elevated mRNA expression of Nrf-2 (1.12 ± 0.14 vs. 1.89 ± 0.12), HO-1 (0.46 ± 0.02 vs. 1.02 ± 0.10), and NQO-1 (1.35 ± 0.09 vs. 1.97 ± 0.18); and elevated protein levels of Nrf-2 (0.92 ± 0.18 vs. 1.43 ± 0.23), Keap1 (0.31 ± 0.03 vs. 0.44 ± 0.02), HO-1 (0.65 ± 0.02 vs. 0.88 ± 0.02), NQO-1 (0.58 ± 0.02 vs. 0.78 ± 0.02), LC3-II/I (0.20 ± 0.004 vs. 0.28 ± 0.01), ATG4D (0.45 ± 0.01 vs. 0.72 ± 0.04), and P62 (0.85 ± 0.01 vs. 0.99 ± 0.03). The *in vitro* experiments further revealed that 3-methyladenine (3-MA) significantly reversed the decreased apoptosis of IEC-6 cells induced by 20 μmol/L SFN (20.60 ± 1.28 vs. 11.50 ± 0.58).

**Conclusion:**

SFN exhibited the protective effect on intestinal mucosa injury after SAH via activating autophagy, which may provide an innovative approach to alleviate the intestinal mucosa injury caused by SAH.

## Introduction

Subarachnoid hemorrhage (SAH) is a life-threatening disease caused by bleeding in the subarachnoid space. Although cerebral vasospasm and brain damage are significant complications that contribute to high mortality rates among patients with SAH (Sheng et al., 2015), gastrointestinal dysfunction also represents a severe complication of this condition (Gambhir et al., 2009). The relationship between gastrointestinal dysfunction and SAH has been documented in several studies (Zhou et al., 2007; Zhao et al., 2016b). It is plausible that stroke or brain trauma may induce abnormal intestinal responses, such as increased intestinal permeability, the overproduction of intestinal cytokines, and translocation of intestinal bacteria and endotoxins (Akhtar and Choudhry, 2011; Phillips et al., 2015). These abnormal intestinal responses not only affect the intestinal mucosa integrity but also influence other tissues and result in multiple organ dysfunction syndrome (MODS) or systemic inflammatory response syndrome (SIRS) (Davenport et al., 1996). Our previous study has shown that SAH induced remarkable intestinal mucosa injury and the overproduction of intestinal cytokines (Zhao and Zhou, 2011).

Sulforaphane (SFN), a naturally occurring isothiocyanate prevalent in cruciferous vegetables such as broccoli and cabbage, demonstrates protective effects against oxidative stress and inflammation through the activation of nuclear factor erythroid 2-related factor 2 (Nrf-2) (Wagner et al., 2010; Hao et al., 2015). Under oxidative or xenobiotic stimuli, Nrf-2 separates from the cytosolic regulatory protein Keap1 and translocates into the nucleus, where it binds to the antioxidant response element (ARE), and regulates a group of antioxidant enzymes to exert protective functions (Shah et al., 2007; Saw et al., 2013). Several studies have reported that Keap1 mediated the regulation of Nrf-2 by SFN, and the Keap1/Nrf-2/ARE signaling pathway was involved in the early brain injury and secondary cognitive impairment following SAH (Liu et al., 2015; Zhao et al., 2016a; Dinkova-Kostova et al., 2017). The Keap1/Nrf-2/ARE signaling pathway is closely related to autophagy (Bartolini et al., 2018). P62, an autophagy adaptor protein, connects autophagy to the Keap1/Nrf-2/ARE signaling pathway (Jiang et al., 2015). Previous studies mainly focused on the involvement of autophagy in brain injury and neural apoptosis following SAH, and enhanced autophagy was also observed in the early stage (Chen et al., 2014; Ho et al., 2018). However, the role of autophagy in SAH-induced intestinal mucosal injury remains unclear.

SFN has been shown to inhibit inflammation and preserve the intestinal mucosal integrity (Zhao et al., 2010; Ohmori et al., 2013). In this study, we aimed to investigate the potential mechanisms through which SFN exerts its protective effects against intestinal mucosal injury in rats following SAH.

## Materials and methods

### Animal preparation

All protocols in this study were approved by the Affiliated Wuxi No. 2 People’s Hospital of Nanjing Medical University. Male Sprague–Dawley (SD) rats (250–300 g) were bought from the Animal Center of Chinese Academy of Sciences (Shanghai, China). The rats were maintained on a standard diet and housed in temperature- and humidity-controlled animal quarters under a 12-h light/dark cycle.

### Rat models of SAH and experimental protocol

The “two-hemorrhage” SAH model of rat was performed as previously described in our other paper (Zhao et al., 2016a). Rats were anesthetized with pentobarbital intraperitoneal injection (40 mg/kg), and the spontaneous respiration was maintained. A small suboccipital incision was made; the occipital bone and the atlanto-occipital membrane were exposed with the aid of a surgical microscope. A 27-gauge needle was used to puncture the atlanto-occipital membrane carefully into the cisterna magna. Fresh autologous nonheparinized blood (0.2 mL) withdrawn from the femoral artery of the same rat was injected into the cisterna magna within 2 min. The bone wax was used to seal the hole to prevent fistula. Then, the incision was immediately sutured after the blood injection. A 30° head-down prone position was maintained for 30 min to ensure adequate blood distribution around the basal intracranial arteries. After 48 h (day 2), the same procedure was repeated. After the operations, the animals were allowed to recover from the effects of anesthesia and returned to their cages. During the period of unconsciousness, the rats were given 2 mL of water every 3 h using a feeding tube.

### Experimental protocol

We divided male SD rats randomly into three groups: Sham + vehicle group (Sham + V, n = 12), SAH + vehicle group (SAH + V, n = 12), and SAH + sulforaphane group (SAH + S, n = 12). Sulforaphane (SFN, Sigma-Aldrich, St. Louis, MO, United States) was dissolved in corn oil (Sigma-Aldrich, St. Louis, MO). A dose of 50 mg/kg in 250–280 μL corn oil was intraperitoneally (IP) injected every 24 h starting from 30 min after the first blood injection. All rats were then followed up with the daily injection of corn oil for 4 days (Zhao et al., 2016a). In the Sham + V group, the same procedures were applied without blood injection, but the same volume of corn oil was injected intraperitoneally. Three days after the second SAH induction (day 5), the animals were euthanized by cervical dislocation.

### Appetite evaluation

Two independent observers, who were unaware of the study’s objectives, recorded the appetite scores after the first SAH induction. The modified appetite-scoring table, as previously described (Zhou et al., 2007), was used to record the scores daily (Table 1).

**TABLE 1:** Appetite scores.

The amount of the food eaten by the animal	Score
1.0	3
>0.5	2
>0.2	1
<0.2	0

### Tissue harvest

The rats were euthanized on day 5 for tissue assays. A 3-cm segment of the mid-ileum was taken and flushed with ice-cold saline. One-half of the segment was stored in liquid nitrogen immediately for enzyme-linked immunosorbent assay (ELISA), RNA reverse transcriptase–polymerase chain reaction (RT-PCR), and Western blot. The other half was immersed in 10% buffered formalin for histopathological studies, including hematoxylin–eosin (HE) staining and ultrastructural observations. Another 3-cm segment of the mid-ileum was taken for the assessment of the intestinal wet/dry weight ratio.

### Hematoxylin–eosin staining

Ileum tissues from each group were fixed in 4% formaldehyde and embedded in paraffin. Deparaffinized sections were sectioned in 5–6 slices with a thickness of approximately 5 μm. These slices underwent routine dewaxing and dehydration processes before HE staining (Abcam, United States) was performed according to established protocols.

### Intestinal wet/dry weight ratio

The wet/dry weight ratio is a reliable index to assess tissue microvascular permeability, and it represents the percentage of water in tissue. The intestinal wet/dry weight ratio was evaluated as described in a previous study (Zhu et al., 2009). Excess fluid was blotted from specimens after the gut tissue samples were taken, and wet weights were measured. Then, the specimens were dried at 80°C for 72 h and re-weighed to measure the weight of the dry content. The gut wet/dry weight ratio was then calculated.

### Inflammatory scores

Inflammatory scores were determined using a scoring system, as follows: epithelium (E): 0, normal morphology; 1, loss of goblet cells; 2, extensive loss of goblet cells in large areas; 3, loss of crypts; and 4, significant loss of crypts in large areas. Infiltration (I): 0, no infiltrate present; 1, infiltration observed around the bases of crypts; 2, infiltration extending to the lamina muscularis mucosa layer; 3, extensive infiltration reaching the muscularis mucosa accompanied by abundant edema; and 4, infiltration into the submucosal layer. The histological score was defined as the sum of these two parameters (total score = E + I).

### Detection of IL-1β, TNF-α, and IL-6 levels in ileum tissues

The frozen ileum tissues were homogenized using a glass homogenizer in 1 mL of the buffer. The buffer was composed of 1 mg/L of pepstatin A, 1 mmol/L of PMSF, 1 mg/L of leupeptin, and 1 mg/L of aprotinin in PBS solution (pH 7.2). Then, the tissue lysates were centrifuged at 12,000 g for 20 min at 4°C. The intestinal levels of inflammatory mediators were quantified using specific ELISA kits for rats, according to the manufacturers’ instructions (TNF-α kit Diaclone Research, France; IL-1β and IL-6 kits, Biosource Europe SA, Belgium). The cytokine levels in the ileum tissue were expressed as the content of cytokine per gram of protein.

### Western blot

The protein levels were quantified as previously described (Zhao et al., 2016a). In brief, protein lysates were prepared from the ileum tissues of rats and rat intestinal epithelial (IEC-6) cells using the precooled RIPA lysis buffer containing the protease inhibitor PMSF; then, they were separated using 12% SDS-PAGE and transferred to nitrocellulose membranes. The membrane was blocked with 5% skimmed milk for 2 h at room temperature and then incubated with primary antibodies at 4°C; the primary antibodies used were as follows: anti-Keap1 (1:500, Santa Cruz Biotechnology, CA), anti-Nrf-2 (1:500, Santa Cruz Biotechnology, CA), anti-HO-1 (1:1,000; Santa Cruz Biotechnology, CA), anti-NQO-1 (1:1,000; Santa Cruz Biotechnology, CA), anti-LC3 (1:2000; Abcam, United States), anti-P62 (1:5,000; Abcam, United States), anti-ATG4D (1:800; Proteintech Group, United States), anti-β-actin (1:1,000, Santa Cruz Biotechnology), and anti-GAPDH (1:1,000, Santa Cruz Biotechnology). Optical densities were obtained using Glyko BandScan software (Glyko, Novato, CA). All experiments were repeated at least thrice.

### RNA reverse transcriptase–polymerase chain reaction

Total RNA was extracted using Trizol (Invitrogen, CA, United States), and cDNA was synthesized from 2 μg of total RNA using the BU-Script RT-Kit (Biouniquer, Jiangsu, China) and stored at −20°C. Reverse transcription was conducted using GoTaq Green Master Mix (Promega, WI, United States). The primers and PCR parameters are shown in Table 2. PCR products were detected through agarose gel electrophoresis. The intensity of the bands was analyzed using the ImageJ program. GAPDH was used as a housekeeping gene.

**TABLE 2:** PCR primer sequences.

Target gene	Sense primer (5’to 3′)	Antisense primer (5’to 3′)	Annealing temperature	Number of cycles	Size (bp)
Nrf2	GGTGATGAATTTTACTCTGC	TTTCCGAGTCACTGATGAACC	55°C	33	278
HO-1	ATCGTGCTCGCATGAACACT	CCAACACTGCATTTACATGGC	57°C	35	339
NQO1	ACTCGGAGAACTTTCAGTACC	TTGGAGCAAAGTAGAGTGGT	53°C	35	492
GAPDH	GTCGGTGTGAACGGATTT	ACTCCACGACGTACTCAGC	56°C	35	276

### Immunofluorescence staining

IEC-6 cells were fixed with 4% of paraformaldehyde and permeabilized with 0.5% Triton X-100 in ice-cold PBS. The pretreated cells were blocked with 1% bovine serum albumin (BSA) and incubated with primary anti-LC3B (1:200; Abcam, United States). The nuclei were stained with 4′, 6-diamidino-2-phenylindole dihydrochloride (DAPI). Images of the stained cells were acquired using a fluorescence microscope (BX41, Olympus, Japan).

### Hoechst 33258 staining

IEC-6 cells were first fixed with fresh 4% paraformaldehyde at 4°C for 45 min and washed using PBS (pH 7.2); then, 0.4% Triton X-100 was used for percolation for 30 min at room temperature. Finally, Hoechst 33258 solution (10 mg/L) was added and placed at room temperature for 5 min in the dark. A fluorescence microscope was used to observe cell morphology. The normal nucleus showed diffused and uniform low-intensity fluorescence, and the nucleus of apoptotic cells showed granular fluorescence.

### Statistical analysis

Statistical analysis of the data was performed using SPSS 12.0. All data were presented as mean ± SD. The Mann–Whitney t-test was used to measure the appetite score from day 0 to day 5. Other measurements were analyzed using one-way analysis of variance and Turkey’s *post hoc* test. We used blinding throughout the data collection and analysis process and corrected for multiple comparisons. The overall significance level was set at 5%.

## Results

### SFN significantly improves appetite and reduces the intestinal wet/dry weight ratio in rats after SAH

**FIGURE 1:**
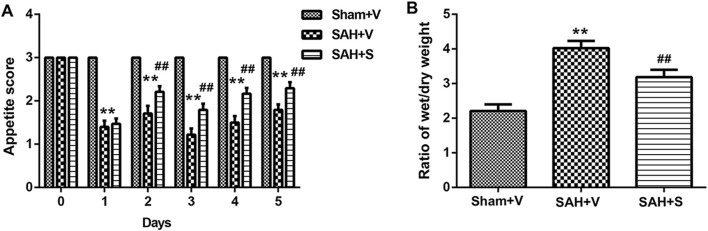
SFN significantly improved appetite and reduced intestinal wet/dry weight ratio in rats after SAH. **(A)** The appetite score of rats in three groups (n = 12, each group) from day 0 to day 5. The modified appetite-scoring table was used to record the scores daily (Table 1). **(B)** The intestinal wet/dry weight ratio of rats in three groups. Data are presented as the mean ± SD. **P < 0.01 vs. Sham + V group; ^##^ *P* < 0.01 vs. SAH + V group.

The rats exhibited a marked loss of appetite after SAH treatment, with the appetite score in the SAH + S group showing significant improvement compared to that in the SAH + V group on days 2, 3, 4, and 5, but still lower than that in the Sham + V group (Figure 1A). The wet/dry weight ratio is a hall marker to determine tissue microvascular permeability. SAH significantly increased the intestinal wet/dry weight ratio at day 5 following SAH, which was markedly decreased by SFN (Figure 1B).

### SFN significantly alleviates ileum tissue injury in rats after SAH

**FIGURE 2:**
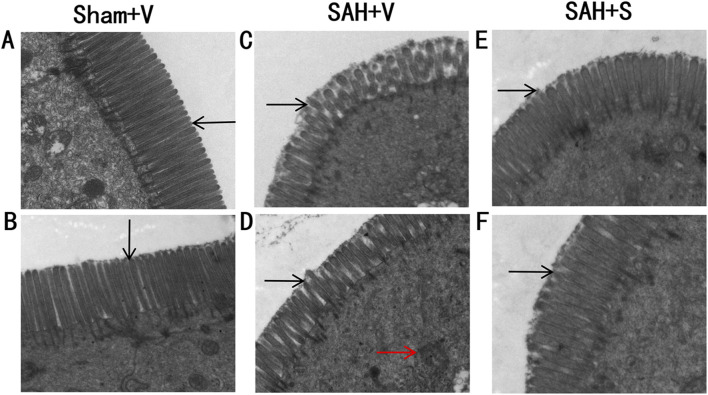
SFN significantly alleviates ileum tissue injury in rats after SAH. Ultrastructural observation of the intestinal mucosa (1 mm^3^) was performed to assess the effect of SFN on ileum tissue injury in rats after SAH. The rats were euthanized on day 5 for tissue assays. Electron microscopic photographs of intestinal mucosal epithelium of rats in the Sham + V group **(A,B)**, SAH + V group **(C,D)**, and SAH + S group **(E,F)** (magnification ×15 k). The black arrows point to microvillous structures. The red arrows indicate the disrupted mitochondria.

Then, special indices such as villous height, crypt depth, villous diameter, and villous surface area were used to evaluate intestinal mucosal damages. As shown in Table 3, compared with the Sham + V group, all these indices were significantly decreased in the SAH + V group. Notably, SFN remarkably increased these indices. Furthermore, ultrastructural observation was performed to assess the effect of SFN on ileum tissue injury in rats after SAH. The structure of microvilli was all arranged in the Sham + V group (Figures 2A,B). However, some ultrastructural alterations, such as ruptured, distorted, and sparse microvilli, were detected in the SAH + V group (Figure 2C). Moreover, the mitochondria reduced, and their cristae were disrupted (Figure 2D). Following SFN administration, the ultrastructural alterations were dramatically attenuated in the SAH + S group (Figures 2E,F).

**TABLE 3:** Histomorphometric changes.

Group	Villous height (μm)	Villous diameter (μm)	Crypt depth (μm)	Surface area (μm)
Sham + V	289.36 ± 4.87	59.79 ± 1.21	79.35 ± 1.37	0.0548 ± 0.00134
SAH + V	176.71 ± 8.73^a^	48.43 ± 1.46^a^	49.64 ± 1.47^a^	0.0321 ± 0.00297^a^
SAH + SFN	218.43 ± 8.38^b^	53.32 ± 1.76^b^	62.57 ± 1.08^b^	0.0416 ± 0.00219^b^

Values are expressed as mean ± SD.

^a^ P <0.05 versus Sham + V group.

^b^ P <0.05 versus SAH + V group.

### SFN significantly reduces intestinal inflammation in rats after SAH

**FIGURE 3:**
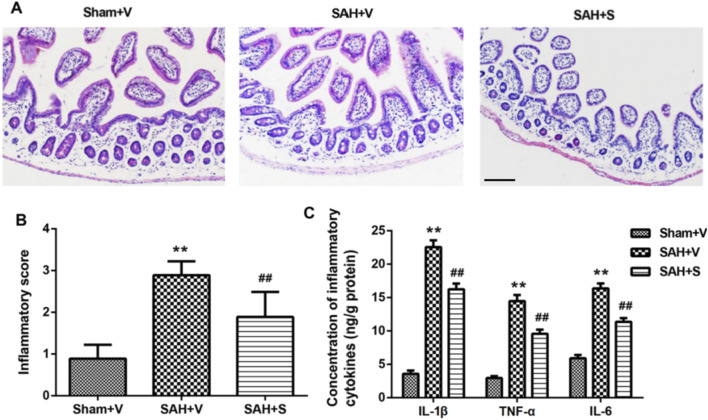
SFN significantly reduced intestinal inflammation in rats after SAH **(A)** Representative histological analysis of ileum tissues in rats via HE staining (scale bar, 100 μm). **(B)** Inflammatory score of ileum tissues in rats. **(C)** The concentrations of inflammatory cytokines (IL-1β, TNF-α, and IL-6) in the ileum tissues of rats were determined using ELISA. Data are presented as the mean ± SD. **P < 0.01 vs. Sham + V group; ^##^ *P* < 0.01 vs. SAH + V group.

HE staining was performed to detect histopathological changes in ileum tissues. Compared with the Sham + V group, the SAH + V group showed significant inflammatory cell infiltration, mucosal interstitial edema, and other pathological phenomena, but SFN treatment significantly improved this effect (Figure 3A), which was further confirmed by the inflammatory score (Figure 3B). In addition, the control group (Sham + V) exhibited relatively low levels of IL-1β, TNF-α, and IL-6 (3.582 ± 0.492, 2.947 ± 0.291, and 5.903 ± 0.487 ng/g protein, respectively) in the ileum tissues, and SAH robustly increased the levels of these cytokines (Figure 3C). Interestingly, SFN administration significantly decreased the upregulated inflammatory cytokines induced by SAH in ileum tissues of rats (Figure 3C).

### SFN significantly upregulates the Keap1/Nrf-2/ARE signaling pathway and autophagy in rats after SAH

**FIGURE 4:**
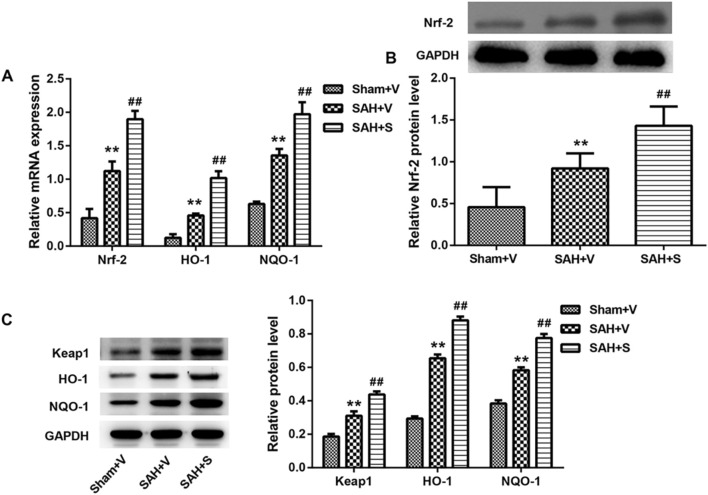
SFN significantly activated the Keap1/Nrf-2/ARE signaling pathway in rats after SAH. **(A)** The mRNA expressions of Nrf-2, HO-1, and NQO1 in the ileum tissues of rats were detected using qRT-PCR. **(B,C)** The protein levels of Keap1, Nrf-2, HO-1, and NQO1 in the ileum tissues of rats were detected using Western blot. Data are presented as the mean ± SD. **P < 0.01 vs. Sham + V group; ^##^ *P* < 0.01 vs. SAH + V group.

**FIGURE 5:**
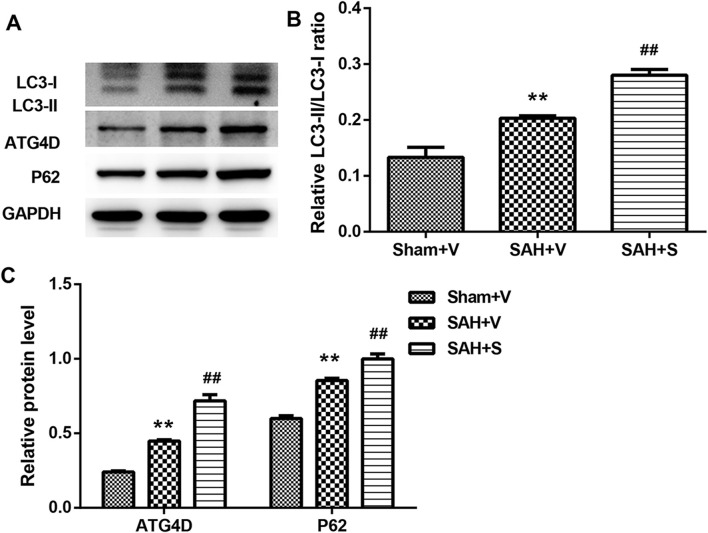
SFN significantly activated autophagy in rats after SAH. **(A–C)** The protein levels of LC3-II, ATG4D, and P62 in the ileum tissues of rats were detected using Western blot. Data are presented as the mean ± SD. **P < 0.01 vs. Sham + V group; ^##^ *P* < 0.01 vs. SAH + V group.

Considering that SFN exhibits the protective effect on oxidative stress and inflammation via the activation of Nrf-2 (Wagner et al., 2010; Hao et al., 2015), here, we tested the Keap1/Nrf-2/ARE signaling pathway using RT-PCR and Western blot. Compared with the Sham + V group, the expressions of Keap1, Nrf-2, HO-1, and NQO-1 were all significantly upregulated in the SAH + V group, and SFN further enhanced the effect of SAH on the Keap1/Nrf-2/ARE signaling pathway, both in mRNA (Figure 4A) and protein levels (Figures 4B,C). Western blot analysis also showed that autophagy-related proteins (LC3-II, ATG4D, and P62) were significantly upregulated in the SAH + V group, and SFN further upregulated the enhanced autophagy in rats after SAH (Figures 5A–C).

### SFN significantly reduces intestinal mucosal epithelial cell apoptosis by regulating autophagy

**FIGURE 6:**
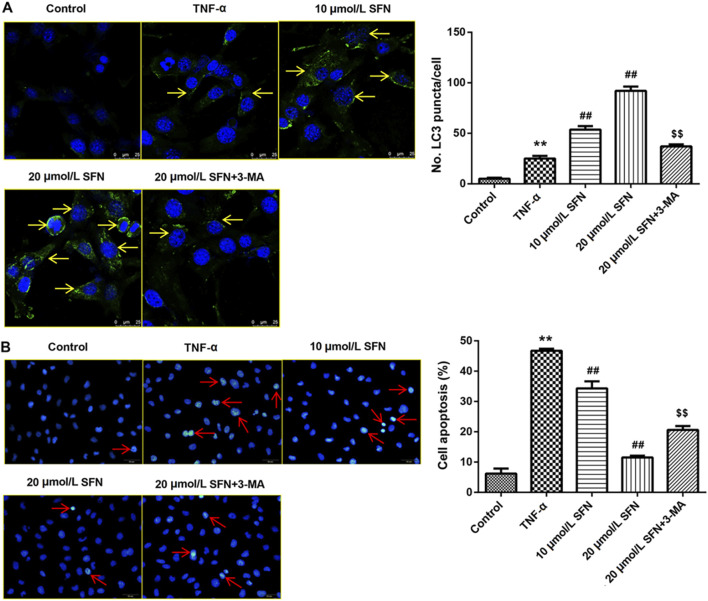
SFN significantly reduced intestinal mucosal epithelial cell apoptosis by activating autophagy **(A)** Punctate distribution of LC3 was examined through immunofluorescence staining. **(B)** Cell apoptosis was examined through Hoechst 33258 staining. Data are presented as the mean ± SD. **P < 0.01 vs. control group; ^##^ *P* < 0.01 vs. TNF-α group; ^$$^ *P* < 0.01 vs. 20 μmol/L SFN group. The yellow arrows indicate the autophagosomes. The red arrows indicate the apoptotic cells.

**FIGURE 7:**
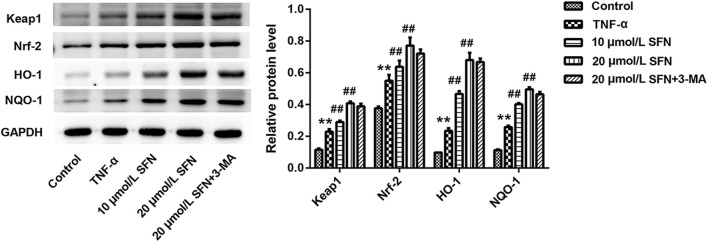
SFN significantly activated the Keap1/Nrf-2/ARE signaling pathway in intestinal mucosal epithelial cells treated with TNF-α. The protein levels of Keap1, Nrf-2, HO-1, and NQO1 in IEC-6 cells were detected using Western blot. Data are presented as the mean ± SD. **P < 0.01 vs. control group; ^##^ *P* < 0.01 vs. TNF-α group.

*In vitro* experiments were conducted to evaluate the role of autophagy in rats after SAH. TNF-α was used in IEC-6 cells to induce cell damage. LC3 immunofluorescence staining showed that TNF-α promoted the formation of autophagosomes, and SFN further upregulated the enhanced autophagosomes in IEC-6 cells after TNF-α treatment (Figure 6A). 3-MA (an inhibitor of autophagy) greatly counteracted the increased autophagosomes induced by SFN (Figure 6A). Moreover, SFN significantly reversed TNF-α-induced apoptosis in IEC-6 cells, and 3-MA dramatically rescued the decreased cell apoptosis induced by SFN (Figure 6B). In addition, SFN further enhanced the upregulated protein levels of Keap1, Nrf-2, HO-1, and NQO-1 induced by SAH in IEC-6 cells, which was similar to the results *in vivo*, whereas 3-MA had no effect on the Keap1/Nrf-2/ARE signaling pathway (Figure 7).

## Discussion

In this study, we demonstrated that SAH-induced tissue edema and inflammatory responses in ileum tissues of rats were significantly attenuated after SFN administration. Both the *in vivo* and *in vitro* experiments showed that the Keap1/Nrf-2/ARE signaling pathway and autophagy were activated in ileum tissues of rats treated with SAH and IEC-6 cells treated with TNF-α. The *in vitro* experiments further showed that SFN significantly upregulated the Keap1/Nrf-2/ARE signaling pathway and autophagy activated by TNF-α. Moreover, SFN also decreased TNF-α-induced cell apoptosis in IEC-6 cells; all of these were dramatically reversed by 3-MA, providing evidence that SFN may protect against SAH-induced intestinal mucosal damages through the activation of autophagy.

Intestinal mucosal injury is a common complication following SAH; however, studies on the structural alterations of intestinal mucosa remain limited. The major changes reported in SAH-induced gastrointestinal abnormity included gut motility dysfunction, stress ulcer, alterations of mucosal absorptive function, and disruption of the gut barrier (Zhou et al., 2007; Davenport et al., 1996). Here, we also observed many damages in the intestinal mucosa, such as the disarrangement of villi, mucosal atrophy, intestinal inflammation, and the fusion of adjacent villi.

Cytokines are pleiotropic and exert different biological activities (Sercombe et al., 2002). Many pro-inflammatory cytokines are cytotoxic, which lead to the destruction of intercellular tight junctions and the increase in the permeability of gut (Faries et al., 1998; Hang et al., 2003). Cytokine-mediated inflammation may be significant in the pathogenesis of structure alterations of intestinal mucosa (Berkes et al., 2003; Chen et al., 2007; Amasheh et al., 2009). In this study, we also noticed the increased inflammatory cytokines (IL-1β, TNF-α, and IL-6), corresponding with our previous findings (Zhao and Zhou, 2011). Our current investigation provides evidence that SAH increases pro-inflammatory cytokines, along with microvascular permeability factors, likely contributing to acute intestinal injuries.

Nrf-2 is a redox-sensitive transcription factor and regulates gene coding for anti-inflammatory, anti-oxidant, and detoxifying proteins (Keum and Choi, 2014; O'Connell and Hayes, 2015). Nrf-2 also activates the expression of several cytoprotective enzymes, such as NQO1 and HO-1, and these products keep the cell away from oxidative, xenobiotic, and inflammatory damages (Wei et al., 2008; Jian et al., 2010). It has been demonstrated that the Keap1/Nrf-2/ARE pathway has a protective effect in various tissues, such as the brain, heart, lung, and intestine (Deng et al., 2013; Kim et al., 2014; Lau et al., 2015). In this study, significantly increased mRNA and protein levels of Keap1, Nrf-2, HO-1, and NQO1 were all found in ileum tissues of rats after SAH, suggesting that the Keap1/Nrf-2/ARE pathway was activated in ileum tissues of rats following SAH. Considering the close relationship between the Keap1/Nrf-2/ARE signaling pathway and autophagy (Bartolini et al., 2018), we examined the protein levels of autophagy-related factors. As expected, there was a significant upregulation in protein levels of LC3-II, ATG4D, and P62 in ileum tissues after SAH. These results indicate a potential involvement of autophagy in tissue injury within the ileum following SAH.

SFN is a potent activator of Nrf-2 and exhibits the protective effect on oxidative stress and inflammation via the activation of the Keap1/Nrf-2/ARE signaling pathway (Wagner et al., 2010; Hao et al., 2015). SFN destroys the cytoplasmic Keap1-Nrf-2 complex by modifying the cysteine residues of Keap1, thus resulting in the release and translocation of Nrf-2 to the nucleus (Itoh et al., 1997; Holland and Fishbein, 2010). In this research, we found that SFN further enhanced the upregulated levels of Keap1, Nrf-2, HO-1, and NQO1 in the ileum tissues of rats after SAH, as well as the upregulated protein levels of LC3-II, ATG4D, and P62. SFN also suppressed inflammation in the ileum tissues of rats after SAH via reducing the release of pro-inflammatory cytokines and ileum tissue injury. More importantly, the *in vitro* experiments revealed that SFN significantly reversed TNF-α-induced cell apoptosis and enhanced the TNF-α-activated Keap1/Nrf-2/ARE signaling pathway and autophagy in IEC-6 cells, which were all reversed by 3-MA.

Due to time limitations and experimental budget constraints, in this experiment, we merely provided simple evidence that SFN can activate the Keap1/Nrf-2/HO-1 signaling pathway. However, we did not elaborate on the underlying mechanism through relevant causal experiments. We will conduct more in-depth experiments and explore the mechanism in subsequent research projects.

## Conclusion

In this study, we demonstrated that SFN significantly improved appetite loss, reduced the intestinal wet/dry weight ratio, and alleviated ileum tissue injury and inflammation of SAH rats. The *in vivo* experiments showed that SFN further enhanced the activated Keap1/Nrf-2/ARE pathway and autophagy in SAH rats. Furthermore, the *in vitro* experiments revealed that the enhanced Keap1/Nrf-2/ARE signaling pathway and autophagy induced by TNF-α were significantly reversed by 3-MA. Above findings provided an innovative approach to alleviate the intestinal mucosa injury caused by SAH.

## Data Availability

The original contributions presented in the study are included in the article/Supplementary Material; further inquiries can be directed to the corresponding author.
