# Correction: The protection of sulforaphane on subarachnoid hemorrhage-induced intestinal mucosa injury in rats

**DOI:** 10.3389/fmolb.2025.1692389

**Published:** 2025-09-17

**Authors:** Zixiang Liu, Pengpeng Li, Yuanhai Zhang, Shidi Zhao, Wei Gao

**Affiliations:** ^1^ Department of Neurosurgery, Jiangnan University Medical Center, Wuxi, Jiangsu, China; ^2^ Department of Neurology, Wuxi Ninth People’s Hospital Affiliated to Soochow University, Wuxi, Jiangsu, China

**Keywords:** SFN, SAH, Keap1/Nrf-2/HO-1 pathway, autophagy, intestinal mucosa injury

Author Wei Gao was erroneously assigned to affiliation Department of Neurosurgery, Jiangnan University Medical Center, Wuxi, Jiangsu, China. This affiliation has now been removed for author Wei Gao.

Affiliation Department of Neurology, Wuxi Ninth People’s Hospital Affiliated to Soochow University, Wuxi, Jiangsu, China was erroneously omitted for author Wei Gao.

The original article has been updated.

